# Older veterans associated with reduced risk of cancer: Retrospective nationwide matched cohort study in Taiwan

**DOI:** 10.3389/fmed.2022.931565

**Published:** 2023-01-04

**Authors:** Li-Fei Pan, Renin Chang, Chung Y. Hsu, Kuan-Hao Tsui

**Affiliations:** ^1^Department of General Affair Office, Kaohsiung Veterans General Hospital, Kaohsiung, Taiwan; ^2^College of Finance and Banking, National Kaohsiung University of Science and Technology, Kaohsiung, Taiwan; ^3^Department of Emergency Medicine, Kaohsiung Veterans General Hospital, Kaohsiung, Taiwan; ^4^Institute of Medicine, Chung Shan Medical University, Taichung, Taiwan; ^5^Graduate Institute of Biomedical Sciences, China Medical University, Taichung, Taiwan; ^6^Institute of Biomedical Sciences, National Sun Yat-sen University, Kaohsiung, Taiwan; ^7^Department of Obstetrics and Gynecology, Kaohsiung Veterans General Hospital, Kaohsiung, Taiwan

**Keywords:** Taiwan, cohort, cancer, veterans, epidemiology

## Abstract

**Importance:**

It remains unknown whether Taiwanese veterans have a lower risk of subsequent cancer compared with non-veterans.

**Objective:**

To examine whether veterans are associated with reduced cancer risk.

**Methods:**

From January 2004 to December 2017, this study included 957 veterans and 957 civilians who were propensity score (PS) matched by years of birth, sex, residence, index year, days in the hospital, frequency of outpatient visits, and relevant comorbidities at baseline. Multivariate Cox proportional hazards regression analysis was applied to compare the risks of cancer, overall and by subgroup, and mortality. All the participants were cancer free at the baseline.

**Exposures:**

Veterans retrieved from Taiwan National Health Insurance Research Database (NHIRD).

**Main outcome:**

Cancer extracted from the Registry for Catastrophic Illness Patients Database (RCIPD).

**Results:**

Overall, 1,914 participants were included, and 957 veterans with a mean (SD) age of 75.9 (6.79) years and 946 men (98.9%). The mean follow-up was about 10.5 (±4.51) years. Cancer was recorded in 6.68% (*N* = 64) and 12.12% (*N* = 116) of veterans and non-veterans, respectively. Veterans were associated with decreased risk [adjusted hazard ratio (aHR), 0.57; 95% CI: 0.41–0.78; *P* < 0.001] of cancer compared with civilians after controlling for age, sex, urbanization, hypertension, diabetes, hyperlipidemia, cardiovascular event, COPD, asthma, chronic liver disease, alcohol-related illness, and Parkinson’s disease. Cancer subgroup analyses verified this finding (HRs <1.0). The decreased incidence rate was predominantly for liver cancer (aHR, 0.18; 95% CI: 0.05–0.72; *P* < 0.05).

**Conclusion:**

Taiwanese older veterans are associated with reduced overall cancer risk than individuals without veteran status.

## Introduction

Cancer is a major global public health issue and the second leading cause of death in the United States, with approximately 5,200 new cases expected to be diagnosed each day in the United States (US) (1). Research has established convincing evidence that much of the risk for cancers can be attributed to extrinsic exposures, with approximately 70–90% of cancer cases due to the patient’s lifestyle behaviors and environmental factors (2). Physically and mentally capable civilians are selected for military service, dedicate time in the military, such as 3–4 years in Scottish, and then leave the service with veteran status. The military population may differ from the general population in terms of exposure to factors associated with cancer risks, such as health behavior, smoking, alcohol consumption, diet, sun exposure, and physical fitness (3). Yet, there is a potential “healthy soldier effect” in which only those who are physically and mentally healthy are selected for the military, and there is evidence that this may carry over into the “healthy veteran effect” (3–6).

Studies of US veterans have shown that the most common malignancies are prostate, lung, and colorectal cancers, similar to trends in the general US population (7). Veterans with diabetes had a higher incidence of liver, pancreatic, and colorectal malignancies than non-diabetic veterans (8). Among Korean veterans who served in the Vietnam War, they have an increased risk of developing overall cancer and several specific cancers in the mouth, salivary glands, stomach, and small intestine (9), but other studies showed inconsistent findings (4, 10). In observational epidemiologic cancer studies, patient interactions with healthcare services, such as frequency of receiving outpatient care, the total length of stay at the baseline, and accessibility of services are important confounding factors (11). However, none of these previous studies included baseline comorbidities and medical accessibility to address detection bias in the analysis of subsequent cancer risk in veterans and non-veterans (4, 9, 10, 12–14).

There are some specific points about Taiwan’s veterans that need to be stated before the study. The most important eligibility issue to become a Taiwanese veteran is that the soldier has completed at least 10 years of military service, which is quite different compared with other countries (5). Veterans in Taiwan did not been through battles or wars that were fraught with the risk of exposure to toxic substances, such as Agent Orange (9, 10), or nerve agent, Sarin (12, 15), which are potentially associated with cancer. Veterans who are not working received a variety of benefits from the Veterans Affairs Council and are exempted from Medicare co-payment. Furthermore, veterans who are homeless or in need of support may be housed in a Veterans’ Home upon proper application. Each Veterans’ Home is geographically integrated into a network of care at a responsible Tertiary Veterans Hospital in Taiwan. Since Taiwanese veterans have a longer length of military service and comprehensive inpatient and discharge support from the national government, we hypothesize that veteran status has a prolonged health effect and lower risk of subsequent cancer development. We conducted this national population-based surveillance cohort study to examine whether participants with veteran status were at risk or protected against the development of cancer.

## Materials and methods

### Data sources

After obtaining approval from the Institutional Review Board (IRB) of the Taiwan Ministry of Health and Welfare, we identified medical records of veterans collected in the National Health Insurance Research Database (NHIRD) between 2000 and 2017. Universal Taiwan’s National Health Insurance (NHI) program administered by the Taiwan Government is single-payer and mandatory and includes >99% of Taiwan’s population (16). NHIRD is available for research purposes with appropriate applications. The validity of NHIRD for use in epidemiological cancer research has been shown in the previous publication (17–24). NHIRD includes patients treated at dedicated veterans’ hospitals. This study was a retrospective evaluation of patient information with no more than minimal risk to the subjects. As it is impossible to identify individual patients, informed consent is not required for this study. The encrypting procedure is identical and the linkage of the claims belonging to the exact patient is constant and feasible for continuous follow-up. The NHIRD records comprehensive and ongoing registration and claims information, including participants’ characteristics, disease diagnoses, outpatient visits, emergency department utility, inpatient information, diagnostic, treatment, operation codes, and prescribed medications. All claims can be linked in chronological order to provide a temporal sequence of all health services utilization. We used five data sources: beneficiary registries, ambulatory care expenditures by outpatient visits, inpatient expenditures by the number of admissions, registry of catastrophic illness patients database, and cause of death data. We used encrypted and unique personal identification numbers to obtain longitudinal medical histories of intake cases. Prior to 2016, the diagnosis codes used in this study were based on the International Classification of Diseases, Ninth Revision, Clinical Modification (ICD-9-CM), and from 2016 onward, the diagnosis codes were based on the ICD-10-CM. This study was approved by the China Medical University Hospital Research Ethics Committee with the certificated number CMUH109-REC2-031 (CR-2).

### Study design and participants

We included people with veteran status as the participants of the study group from January 2004 to December 2015 and follow-up until 2017. The veteran status is determined with a code of the Registry for Beneficiaries in the NHIRD to identify ID1_UNIT = 61. The study database contains 2 million people, which was sampled by BNHI from the original claim data of NHIRD. There was no significant difference in the sex (*P* = 0.613) and age distribution between the subset data and the original NHIRD. The duration of 2000–2003 was set to be the baseline period to collect demographic and comorbidities information, and this period to be the look-back period to ensure all participants in both groups are free of cancer before this study. The reference group included patients who never had the status of veterans during the follow-up period. We matched one control civilian from the general population to each individual with veteran status on age, sex, and urbanization level of residence (i.e., the information about urbanization is based on the participants’ urbanization level of residence; level 1 denotes most urbanized and level 4 denotes least urbanized. This is a proxy for healthcare availability), index year, baseline disease severity (proxy by days in the hospital and frequency of outpatient visits at the baseline), and relevant comorbidities. To mitigate potential immortal time and survival bias, the index year was set 3 years after all participants of both groups entering NHIRD and the follow-up started subsequently. Control participants were alive on the index year. To estimate the risk of cancer, both groups were followed from the index year until the first cancer was diagnosed, death, or the end of the observation period (31 December 2017), whichever came first. The exclusion criteria were patients with missing information on age and sex, diagnosis of cancer before the index date, and a follow-up time of less than 1 year.

### Outcome variables: Overall, specific cancer, and all-cause mortality

The outcome variables were overall cancer and prevalent specific cancer, ascertained by linking the unique identification number of each participant to the Registry for Catastrophic Illness Patients Database (RCIPD). Codes from the *International Classification of Diseases, Ninth Revision Clinical Modification* (ICD-9-CM), and *Tenth Revision (ICD-10)* were used to identify cancer. The positive predictive rate was 93% (19). Each participant was followed up until a diagnosis of cancer (ICD-9-CM code 140-208 and ICD-10-CM C00-C96) was made. We set all-cause mortality as the secondary outcome to examine the potential competing status of death on cancer.

### Covariates

The patient’s age and sex were recorded at the index year. Baseline comorbidities were extracted from at least three consistent medical claims records prior to the index year (i.e., 2000–2003). We identified the presence of the following comorbidities using ICD-9-CM codes: hypertension (ICD-9-CM codes 401-405), diabetes (ICD-9-CM code 250), hyperlipidemia (ICD9-CM code 272), cardiovascular disease (CVD) (ICD9-CM code 414-410 and 430-430), chronic obstructive pulmonary disease (COPD) (ICD-9-CM code 490-492 and 493-496), asthma (ICD-9-CM code 493), chronic hepatitis (ICD-9-CM code 571.4), chronic kidney diseases (CKD) (ICD-9-CM code 585), and Parkinson’s disease (PD) (ICD-9-CM code 332.0). To address detection bias, we included the length of hospital stay and frequency of outpatient department within 1 year before the index year into the study.

### Statistical analyses

Baseline characteristics of participants are shown as event numbers and percentages for categorical variables and as means and standard deviations for continuous variables. Chi-square was adopted to examine the categorical variables and Student’s *t*-test for continuous variables. The matching policy in our study was individual propensity score matching rather than frequency matching. The veteran group was matched to the non-veteran group based on their propensity score (1:1 ratio) using the nearest neighbor matching process, initially for the eighth digit, and then for the first digit as needed. Thus, matches were first made within a caliper width of 0.0000001, and then the caliper width was gradually increased to 0.1 for unmatched cases. In this way, some variables may remain different between the two groups, such as the urbanization level in this study. The crude hazard ratio (cHR) was estimated by the univariable Cox proportional hazards model, and then repeated with adjustment for the relevant confounding effect of covariates. The incidence of cancer during follow-up was calculated by dividing the number of events by the respective person-years at risk and presented as the number of events per 1,000 person-years. Kaplan–Meier curve was used to describe the cumulative incidence of overall cancer in the study and reference groups with the log-rank test. We used multivariable Cox proportional hazards regression models to examine the potential effect of veteran status on the cumulative risk of cancer after adjustment for age, sex, and covariates list in Table 1 including hypertension, diabetes, hyperlipidemia, CVD, COPD, asthma, chronic liver disease, CKD, and PD. Schoenfeld residual analysis was adopted to confirm the assumption of no violation of proportionality risk. Analyses were performed using SAS statistical software version 9.4 (SAS Institute Inc.). All the tests of significance were two-tailed with a *P*-value of <0.05 was considered statistically significant. Cox proportional hazards analysis was used to compare the risks of death between veterans and non-veterans.

**TABLE 1:** Demographic characteristics of groups with and without veteran status.

Variable	Unmatch veteran status				*P*-value	After PS match veteran status				*P*-value
	No (*N* = 1,250,337)		Yes (*N* = 963)			No (*N* = 957)		Yes (*N* = 957)		
	*n*	%	*n*	%		*n*	%	*n*	%	
Age mean ± SD (years)^b^	43.6 ± 15.8		75.9 ± 6.80		<0.001	76.3 ± 8.00		75.9 ± 6.79		0.19
Birth year					<0.001					0.001
1917–1926	20,933	1.67	528	54.8		562	58.7	524	54.8	
1927–1936	64,461	5.16	331	34.4		250	26.1	331	34.6	
Others	1,164,943	93.2	104	10.8		145	15.2	102	10.7	
Sex					<0.001					0.02
Male	625,656	50.0	952	98.9		932	97.4	946	98.9	
Female	624,681	50.0	11	1.14		25	2.61	11	1.15	
Urbanization^c^					<0.001					<0.001
1	694,315	55.5	500	51.9		386	40.3	497	51.9	
2	452,655	36.2	236	24.5		431	45.0	235	24.6	
3	87,324	6.98	172	17.9		111	11.6	170	17.8	
4	16,033	1.28	55	5.71		29	3.03	55	5.75	
Length of hospital stay^a^	0.94	26.2	11.9	106.9	<0.001	17.8	302.9	11.9	107.2	0.57
Frequency of ambulatory use^a^	14.1	15.8	29.3	28.3	<0.001	27.2	31.5	27.2	27.6	0.86
**Comorbidity**										
Hypertension	281,699	22.5	692	71.9	<0.001	663	69.3	686	71.7	0.25
Diabetes	134,576	10.8	250	26.0	<0.001	223	23.3	247	25.8	0.20
Hyperlipidemia	225,743	18.1	148	15.4	<0.001	134	14.0	148	15.5	0.37
Cardiovascular disease	145,140	11.6	514	53.4	<0.001	515	53.8	509	53.2	0.78
COPD	97,632	7.81	551	57.2	<0.001	532	55.6	545	57.0	0.55
Asthma	64,279	5.14	117	12.2	<0.001	116	12.1	116	12.1	0.99
Chronic liver diseases	72,045	5.76	22	2.28	<0.001	18	1.88	22	2.30	0.52
Chronic kidney disease	5,077	0.41	19	1.97	<0.001	22	2.30	19	1.99	0.64
Parkinson’s disease	9,290	0.74	59	6.13	<0.001	63	6.58	58	6.06	0.64
Follow time for cancer mean ± SD (years)	9.94 ± 4.29		10.5 ± 4.51		0.003	10.5 ± 4.53		10.5 ± 4.51		0.91
Follow time for death mean ± SD (years)	10.0 ± 4.29		10.6 ± 4.48		<0.001	10.8 ± 4.54		10.6 ± 4.48		0.32

^a^Data traced back 1 year before the index date.

^b^Student’s *t*-test.

^c^Urbanization level of residence (levels 1–4, most to least urbanized), a proxy for healthcare availability in Taiwan.

### Subgroup analyses

We stratified the data by year of birth in our analysis, dividing the data into 10-year bands to improve statistical power and examine potential birth cohort effects. We conducted a subgroup analysis to assess the association of the presence or absence of comorbidity with cancer in terms of veteran status. Furthermore, we determined the potential effect of veteran status on the risk of new-onset specific cancer type. Cancer type of the small number of subjects will not be shown in our report for the political “Personal Information Protection Act.” Patients with diabetes are at risk for cancer, thus, we conducted a further subgroup analysis based on the status of diabetes or not among veterans in our study.

### Patient involvement data availability statement

The data source used in this study was the claims data of NHIRD published by Taiwan National Health Insurance. Participants were not involved in the retrospective secondary cohort study. For Taiwan legal restrictions according to the “Personal Information Protection Act,” data in this study cannot be made publicly available. Data requests for revision can be made as a formal appointment according to the original proposal.

## Results

A total of 1,914 participants were enrolled between January 2004 and December 2015. Among them, we identified the veteran group (*N* = 957) and the non-veteran control group (*N* = 957) after propensity score matching. We selected controls based on strict criteria of the same age, sex, index year, and comorbidities distribution.

As shown in Table 1, 98.9% of the veterans were male and 54.8% of them were born between 1917 and 1926. Gender differences were consistent with the gender distribution of the armed forces. Although the veteran group presented with more comorbidities such as hypertension, CVD, COPD, and asthma compared with the non-veteran group before the propensity score (PS) match. These comorbidities and frequency of medical utility were well-balanced after the PS match (Table 1, right column). The mean (SD) age was 75.9 (6.79) years. The mean (SD) follow-up time for cancer was 10.5 (4.51) years. The mean (SD) follow-up time for death was 10.6 (4.48) years. Although not all variables between the two cohorts were matched to statistically insignificant, we used the multivariate Cox proportional hazards regression model to adjust these variables.

The association between veteran status and cancer risk was shown in Table 2. By the end of the study, 64 (6.68%) veterans had a medical record of cancer diagnosis compared with 116 (12.12%) non-veterans. The veteran group had a lower incidence rate (IR) of cancer (IR, 6.38 per 1,000 person-years) than those in the control group (IR, 11.5 per 1,000 person-years). After adjustment for birth year, sex, urbanization, and comorbidities, the adjusted HR (aHR) of cancer for the veteran group relative to the control group was 0.57 [95% confidence interval (CI): 0.41–0.78; *P* < 0.001]. Among the comorbidities, the risk of cancer in participants with diabetes was 1.47 times greater than those without diabetes (95% CI: 1.04–2.07; *P* = 0.03). The risk of cancer in participants with chronic liver disease was 2.22 times greater than those without chronic liver disease (95% CI: 1.01–4.88; *P* = 0.046).

**TABLE 2:** Cox model measured hazard ratio and 95% confidence intervals of cancer associated with and without veteran status and covariates on the propensity score matched cohorts.

Characteristics	Event no. (*n* = 180)	Person-years	IR^#^	Crude	Adjusted^†^
				**HR (95% CI)**	**HR (95% CI)**
**Veteran status**					
No	116	10,051	11.5	Ref.	Ref.
Yes	64	10,029	6.38	0.56 (0.41, 0.76)***	0.57 (0.41, 0.78)***
**Birth year**					
1917–1926	97	11,017	8.80	1.21 (0.73, 2.01)	1.17 (0.70, 1.95)
1927–1936	65	6,593	9.86	1.30 (0.77, 2.19)	1.30 (0.77, 2.22)
1947–1976	18	2,469	7.29	Ref.	Ref.
**Sex**					
Male			4.82	2.00 (0.50, 8.06)	2.25 (0.55, 9.20)
Female			9.05	Ref.	Ref.
**Urbanization^a^**					
1	91	9,255	9.83	Ref.	Ref.
2	61	6,965	8.76	0.88 (0.64, 1.22)	0.78 (0.56, 1.08)
3	22	2,887	7.62	0.78 (0.49, 1.24)	0.86 (0.54, 1.37)
4	6	973	6.17	0.61 (0.27, 1.39)	0.60 (0.26, 1.38)
**Comorbidities**					
Hypertension			9.43	1.24 (0.90, 1.71)	1.20 (0.83, 1.74)
Diabetes			11.5	1.44 (1.04, 1.98)*	1.47 (1.04, 2.07)*
Hyperlipidemia			9.92	1.10 (0.74, 1.63)	0.89 (0.58, 1.36)
Cardiovascular disease			9.56	1.21 (0.90, 1.62)	1.16 (0.84, 1.61)
COPD			8.63	0.97 (0.73, 1.30)	0.89 (0.65, 1.23)
Asthma			11.0	1.27 (0.82, 1.97)	1.42 (0.89, 2.26)
Chronic liver diseases			19.0	2.27 (1.07, 4.83)*	2.22 (1.01, 4.88)*
Alcohol-related illness			7.23	0.81 (0.20, 3.26)	0.96 (0.23, 3.93)
Parkinson’s disease			3.64	0.42 (0.16, 1.13)	0.42 (0.15, 1.13)

IR^#^, per 1,000 person-years; crude HR represented relative hazard ratio; adjusted HR^†^ represented adjusted hazard ratio: mutually adjusted for birth year, sex, urbanization, and comorbidities in Cox proportional hazard regression. ^a^Urbanization level of residence (levels 1–4, most to least urbanized), a proxy for healthcare availability in Taiwan.

*p < 0.05, ***p < 0.001.

Table 3 shows the results of the subgroup analysis. After adjustment for birth year, sex, urbanization, and comorbidities, veteran status was not associated with an increased risk of cancer development (all HR <1). The age-specific aHR of cancer was lowest for a patient with the birth year of 1917–1926 (aHR, 0.46; 95% CI: 0.30–0.73; *P* < 0.001). The aHRs of cancer for the veteran to the non-veteran group were universally reduced; aHR, 0.35 (95% CI: 0.13–0.89) in all participants without comorbidities and 0.61 (95% CI: 0.43–0.85) in all participants with comorbidities. Specifically, the risk of subsequent cancer in veterans was lower in the subgroup without diabetes (aHR 0.51; 95% CI: 0.35–0.75), but the potential protective effect appeared to be diminished in the subgroup with diabetes (aHR, 0.68; 95% CI: 0.38–1.21).

**TABLE 3:** Comparison of incidence and hazard ratio of cancer stratified by birth year and comorbidity between groups with and without veteran status on the propensity score matched cohorts.

	Without veteran status			With veteran status				
Variable	Event	PY	IR^#^	Event	PY	IR^#^	Crude HR (95% CI)	Adjusted HR^†^ (95% CI)
**Birth year**								
1917–1926	67	5,595	12.0	30	5,423	5.53	0.46 (0.30, 0.71)***	0.46 (0.30, 0.73)***
1927–1936	36	3,014	12.0	29	3,579	8.10	0.71 (0.44, 1.16)	0.78 (0.47, 1.30)
1947–1976	13	1,443	9.01	5	1,027	4.87	0.54 (0.19, 1.52)	0.60 (0.20, 1.80)
**Comorbidity^‡^**								
No	17	1,562	10.9	6	1,523	3.94	0.37 (0.14, 0.93)*	0.35 (0.13, 0.89)*
Yes	99	8,489	11.7	58	8,506	6.82	0.59 (0.43, 0.81)**	0.61 (0.43, 0.85)***
**Diabetes**								
No	84	7,821	10.7	43	7,630	5.64	0.53 (0.36, 0.76)***	0.51 (0.35, 0.75)***
Yes	32	2,230	14.4	21	2,399	8.75	0.62 (0.36, 1.08)	0.68 (0.38, 1.21)

IR^#^, per 1,000 person-years; crude HR represented relative hazard ratio; adjusted HR^†^ represented adjusted hazard ratio: mutually adjusted for birth year, sex, urbanization, and comorbidities in Cox proportional hazard regression. ^‡^Patients with any comorbidity of hypertension, diabetes, hyperlipidemia, cardiovascular disease, COPD, asthma, chronic liver diseases, alcohol-related illness, and Parkinson’s disease was defined as the comorbidity group.

*p < 0.05, **p < 0.01, ***p < 0.001.

Table 4 shows the comparison of the IR of cancer type between the veteran group and the non-veteran control group. The data illustrate that veteran status was significantly inversely associated with liver cancer (aHR, 0.18; 95% CI: 0.05–0.72; *P* < 0.05). There was no significant impact of veteran status on the risk of cancer, including stomach cancer (aHR, 0.76; 95% CI: 0.28–2.05), lung cancer (aHR, 0.90; 95% CI: 0.46–1.76), prostate cancer (aHR, 0.84; 95% CI: 0.36–1.96), and kidney and bladder cancer (aHR, 0.43; 95% CI: 0.14–1.40). There was a reduced risk for colorectal cancer with crude HR 0.46 (95% CI: 0.22–0.97) and almost achieved statistical significance, aHR 0.49, 95% CI: 0.23–1.05, although the number of cases was small.

**TABLE 4:** Comparison of incidence and hazard ratio of cancer type on the propensity score matched cohorts.

	Veterans					
	No		Yes			
Outcome	Event	IR^#^	Event	IR^#^	Crude HR (95% CI)	Adjusted HR^†^ (95% CI)
Stomach cancer	10	0.99	7	0.70	0.71 (0.27, 1.87)	0.76 (0.28, 2.05)
Liver cancer	15	1.49	3	0.30	0.20 (0.06, 0.70)*	0.18 (0.05, 0.72)*
Lung cancer	20	1.99	17	1.70	0.85 (0.45, 1.63)	0.90 (0.46, 1.76)
Colorectal cancer	22	2.19	10	1.00	0.46 (0.22, 0.97)*	0.49 (0.23, 1.05)
Prostate cancer	12	1.19	11	1.10	0.93 (0.41, 2.10)	0.84 (0.36, 1.96)
Kidney and bladder	11	1.09	4	0.40	0.37 (0.12, 1.15)	0.43 (0.14, 1.40)

IR^#^, per 1,000 person-years; crude HR represented relative hazard ratio; adjusted HR^†^ represented adjusted hazard ratio: mutually adjusted for birth year, sex, urbanization, and comorbidities in Cox proportional hazard regression.

*p < 0.05.

Table 5 shows the comparison of the IR of death and estimation stratified by birth year and comorbidities. The risk of death in veterans and non-veterans did not differ significantly [aHR, 1.03; (95% CI: 0.93–1.14)]. In the age subgroup (born in 1917–1926 and 1937 onward), the aHR of all-cause mortality was 0.97 (95% CI: 0.85–1.11) and 1.09 (95% CI: 0.80–1.49), respectively. In the age subgroup (born in 1927–1936), the aHR of all-cause mortality was 1.51 (95% CI: 1.22–1.88).

**TABLE 5:** Comparison of incidence and hazard ratio of all-cause mortality and stratified by birth year.

Variable	Veterans						Crude HR (95% CI)	Adjusted HR^†^ (95% CI)
	No			Yes				
	Event	PY	Rate^#^	Event	PY	Rate^#^		
All	722	10358	69.7	743	10164	73.1	1.06(0.96, 1.18)	1.03(0.93, 1.14)
Birth year								
1917–1926	474	5732	82.7	427	5476	78.0	0.93(0.81, 1.06)	0.97(0.85, 1.11)
1927–1936	146	3153	46.3	239	3659	65.3	1.53(1.25, 1.89)***	1.51(1.22, 1.88)***
Others	102	1473	69.2	77	1029	74.8	1.08(0.80, 1.45)	1.09(0.80, 1.49)

IR^#^, per 1,000 person-years; crude HR represented relative hazard ratio; adjusted HR^†^ represented adjusted hazard ratio: mutually adjusted for birth year, sex, urbanization, and comorbidities in Cox proportional hazard regression.

***p < 0.001.

**FIGURE 1:**
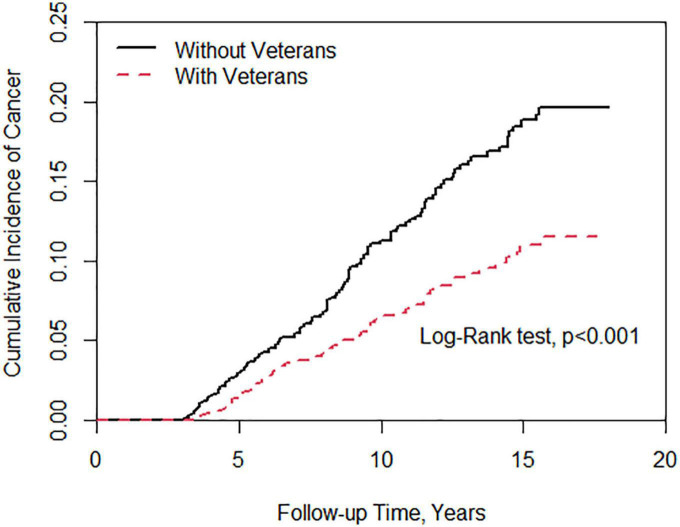
Cumulative incidence of cancer between individuals with and without veteran status.

**FIGURE 2:**
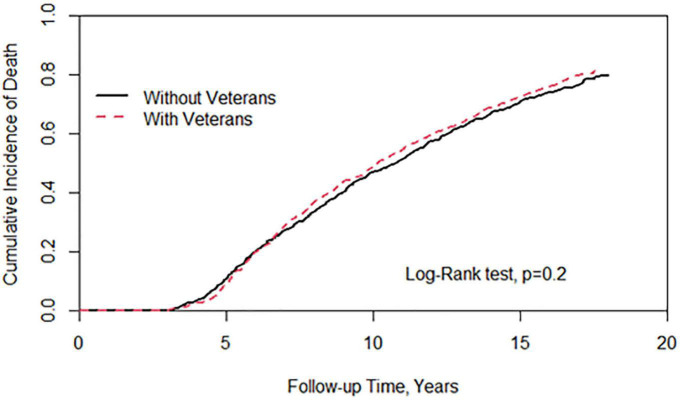
Cumulative incidence of all-cause mortality between individuals with and without veterans status.

Figure 1 showed a statistically significant difference in the risk of cancer between patients with veteran status and those without veteran status. There was a reduction in cancer risk in the veteran group (log-rank test, *P* < 0.001). Figure 2 demonstrates no significant difference in the risk of death between the two groups.

## Discussion

To the best of our knowledge, this is the first countrywide large-scale study to investigate the association between veteran status and cancer in East Asians. Our study concludes that older military veterans (those born in 1917–1926) were at reduced risk of cancer in comparison with civilians without veteran status in Taiwan, after matching for birth year, sex, geographical region, medical accessibility, and comorbidities. The risk of all cancer among younger veterans (those born 1927 onward) did not have an increased risk experienced by non-veterans (all HR <1). Increased cancer risk in patients with diabetes and chronic liver disease were consistently reported in previous studies (25).

One study of the Korean veteran’s population described a higher incidence of lung, bladder, kidney, prostate, and colon cancer than in the general population (10). In another study on New Zealand Vietnam War veterans, the authors demonstrated that overall cancer risk was not significantly increased but hematologic malignancies (4). A recent study demonstrated that after considering the healthy worker effect, veterans exposed to herbicides decades ago have a higher risk of all cancer and an increased incidence of the esophagus, lung, liver, and gastric malignancies (9). Chemical exposure, such as tactical herbicides and Agent Orange, was a significant feature of the previous study. However, veterans in Taiwan did not attend Vietnam or Gulf War during their military service, and the overall risk of exposure to a human carcinogen, such as herbicides, is less likely. In addition, the previous study mentioned that US veterans have poorer health behaviors, such as current heavy alcohol and tobacco consumption (3), which have a role in cancer development (14). Although precise information on differences in chemical exposure and healthy lifestyle between Taiwan and other countries is not available, it is reasonable to presume that such disparity contributed in part to the almost opposite results of our study and previous cohorts in terms of veteran status and cancer risk. The reduction in cancer incidence in our study may be due to the fact that the effects of healthy soldiers were not offset by human carcinogen exposure in Taiwan.

Our study demonstrates the difference in overall cancer risk between veteran personnel and the general population. Incidence rates were lower in veteran personnel than in the general population for stomach, liver, lung, colorectal, prostate, kidney, and bladder cancer. But the differences in rates between the two groups were significant only for liver cancer. It is unclear why people with veteran status in Taiwan have a lower risk of cancer than other civilians, and the factors that may be associated with this difference are outlined below. Men who enter the military and leave as veterans are a specific group because those with certain diseases or conditions are ineligible for military service. In a 2015 national report, the prevalence of hepatitis B virus in Taiwan was 15–20%, with about 2.5 million adult carriers. We speculate that veterans are more likely to have been vaccinated against hepatitis, which is strongly associated with liver cancer, so the risk of liver cancer is significantly lower in those with veteran status than those without veteran status. One report noted a slight increase in the prevalence of smoking during military service and an even higher prevalence of smoking among younger military personnel compared with the general population (26). After 10 years of military anti-smoking health promotion programs, there have been some beneficial effects in reducing smoking rates among Taiwanese military personnel (27). However, the prevalence of smoking among veterans remains unclear and requires further investigation. Due to policy on developments of veteran health promotion and free access to medical care, the opportunity of veterans to receive adequate cancer screen is no less than the civilians. On the other hand, the all-cause mortality is no more than the civilians, which has been shown in our results in Tables 1, 5. Although the results were inconsistent with the previous studies (8, 15), our study used a more stringent approach, which included frequency of medical use and baseline comorbidities to avoid measurable confounding and detection bias, providing a more rigorous conclusion.

### Limitations

First, the specific biological basis of the retrospective cohort study is speculative. To minimize the observational confounding, we matched relevant comorbidities between the two groups at the baseline, which were not provided in the previous studies (4, 7, 8, 10, 13–15, 25). However, the urbanization level was not well-matched in this study. There is still a possibility of selection bias. Fortunately, we used this single-payer insurance health database with a well-validated coding for the outcome covariates (19), and this study was able to overcome the possible bias, and thus indicate a meaningful association.

Second, NHIRD cannot provide information on the dates the veteran entered and left the military, the battles in which they were involved, or the Navy, Army, or Air Force to which the veteran belonged at the time of initial service. However, randomized clinical trials are not feasible because there are different models of military services such as compulsory and voluntary soldiers. A major concern is the age of the study subjects. With a mean age of 75.9 years at the baseline, any potential subjects (veterans and non-veterans) who developed cancer in middle age will not have been captured as they will have been left-truncated either by mortality or by the exclusion of those who already had a cancer diagnosis at the baseline. Thus, both the veterans and non-veterans represent a “survivor population” at the baseline. This has the potential to introduce bias if the incidence of earlier-onset cancer differed between veterans and non-veterans.

Third, any issue of a small number of subjects (less than 3) will not be shown in our report for political restrictions according to the “Personal Information Protection Act” in Taiwan. Therefore, data and interpretation of female veteran-associated cancers are not available in this study.

There were other residual confounders in this study, such as obesity, alcohol, tobacco consumption, diet, exercise, and health behaviors. After accounting for the known limitations of observational studies, our results alleviate the public’s concern about the cancer risk profile of veterans after military service, however, the number of cases of cancer was small.

## Conclusion

In the study, we provide assurance that older individuals with veteran status have a lower risk of developing cancer compared with those without veteran status. A more comprehensive study encompassing a larger cohort and a longer follow-up period is warranted.

## Data availability statement

The raw data supporting the conclusions of this article will be made available by the authors, without undue reservation.

## Ethics statement

This study was approved by the China Medical University Hospital Research Ethics Committee with certificated number CMUH109-REC2-031(CR-2). Written informed consent for participation was not required for this study in accordance with the national legislation and the institutional requirements.

## Author contributions

L-FP, RC, and K-HT: study conception and design, analysis and interpretation of data, and investigation. RC and CH: acquisition of data. L-FP and RC: writing—original draft preparation. CH, RC, and K-HT: writing—review and editing. All authors contributed to the article and approved the submitted version.
